# Celiac Disease: A Forty-Year Analysis in an Italian Referral Center

**DOI:** 10.3390/nu16142292

**Published:** 2024-07-17

**Authors:** Lisa Lungaro, Anna Costanzini, Francesca Manza, Fabio Caputo, Francesca Remelli, Stefano Volpato, Roberto De Giorgio, Umberto Volta, Giacomo Caio

**Affiliations:** 1 Department of Translational Medicine, St. Anna Hospital, University of Ferrara, 44124 Ferrara, Italy; lisa.lungaro@unife.it (L.L.); anna.costanzini@unife.it (A.C.); mnzfnc@unife.it (F.M.); fabio.caputo@unife.it (F.C.); 2 Department of Medical Science, University of Ferrara, 44124 Ferrara, Italy; rmlfnc1@unife.it (F.R.); stefano.volpato@unife.it (S.V.); 3 Geriatrics Unit, Azienda Ospedaliero-Universitaria of Ferrara, 44124 Ferrara, Italy; 4 Department of Medical and Surgical Sciences, University of Bologna, 40138 Bologna, Italy; 5 Mucosal Immunology and Biology Research Center, Massachusetts General Hospital-Harvard Medical School, Boston, MA 02114, USA

**Keywords:** celiac disease, clinical features, natural history, osteoporosis, refractory celiac disease

## Abstract

Background: Celiac disease (CD) is an autoimmune disorder triggered by gluten ingestion. Herein, we assessed clinical, serological and histopathological findings of a single-center, large cohort of CD patients diagnosed and followed-up over forty years. Methods: From January 1980 to December 2020, 1547 CD patients (1170 females; age range: 8–81 years; F:M ratio = 3.1:1) were diagnosed in an Italian tertiary referral center. Comorbidities and complications were recorded at diagnosis and during follow-up. Results: CD diagnoses quadrupled after 2000. The most frequent phenotype was the non-classical CD (63.3%), and the most prevalent histotype was Marsh 3C (44.7%). Gastrointestinal manifestations, detectable in 51% of patients, were diarrhea (24.3%), bloating (28%) and aphthous stomatitis (19.7%). The most common CD-associated disorder was osteopenia (59.9%), predominant in females (64.3%); extraintestinal manifestations included anemia (35.8% iron-deficiency; 87% folic acid malabsorption), cryptogenic hypertransaminasemia (27.9%), and recurrent miscarriages (11.5%). Thyroiditis (26.9%), type 1 diabetes mellitus (2.9%), and dermatitis herpetiformis (1.4%) were the most common CD-related autoimmune disorders. Six patients had inflammatory bowel disease. Complications and mortality rate occurred in 1.8% and 1.9%, respectively. Conclusions: This single-center, large cohort analysis confirmed that CD presentation changed over the years, with an increase of non-classical and subclinical clinical phenotypes.

## 1. Introduction

Celiac disease (CD) is a gluten-related autoimmune enteropathy occurring in genetically predisposed individuals mainly exhibiting human leukocyte antigen (HLA) DQ2 or DQ8 [1]. The disease onset can occur at any age, with a broad range of symptoms clinically categorized as symptomatic (i.e., further distinct in “classical” and “non-classical”) and subclinical (i.e., “subclinical”) phenotypes, according to the Oslo classification [2]. Although the timing and mechanisms promoting the disease onset are still poorly understood, some factors (e.g., gastrointestinal infections, high-fat/high-sugar diet, and microbiota changes) are known to be involved in the development of CD [3,4,5,6].

The discovery of new diagnostic tools (i.e., serological markers) has broadened the knowledge of CD over the years, changing its clinical profile from a rare pediatric food intolerance leading to villous atrophy and food malabsorption to a frequent disorder likely affecting more than 1% of the worldwide population [1,7]. Over the last 20 years, highly predictive serological tests, including antibodies to endomysium (EmA) and TG2 (anti-TG2) [8], and deamidated gliadin peptide (DGP) reduced diagnostic delay and improved CD detection, thus identifying patients with very different symptoms from classical (e.g., diarrhea) to unexpected ones (e.g., extra-intestinal manifestations) or even asymptomatic [7]. However, the high variability of CD clinical presentation and the different serological and histological patterns of the disease necessitate constant observation of CD changing clinical profile to obtain a more appropriate and comprehensive definition of the disease and its multifaceted aspects. The current investigation aims to broaden knowledge of the constant evolution of the disease pathology.

In this retrospective study, we analyzed 1547 consecutive adult CD patients diagnosed in a single Italian referral center for 40 years, from 1980 to 2020. Specifically, we aimed to: (i) determine the most common phenotype and histotype at the time of diagnosis; (ii) define the change of their prevalence over the years; (iii) establish whether a determined phenotype/histotype could influence the disease outbreak; and (iv) evaluate a possible histotype-phenotype correlation and whether CD-associated disorders can correlate with the clinical phenotype. Finally, we assessed the serology and the occurrence of complications associated with CD.

## 2. Materials and Methods

This is a retrospective study on 1547 CD patients (1170 females and 377 males; mean age at diagnosis: 33.1 ± 24.4 years; F:M ratio = 3.1:1; age range: 8–81 years), consecutively diagnosed from January 1980 to December 2020 at our referral center (Department of Internal Medicine, St. Orsola-Malpighi Hospital, Bologna, Italy). Written informed consent was obtained from all recruited patients, who consented to publish their data. Since patients were not individually identified, a simplified International Review Board approval by the Ethics Committee of the St. Orsola Malpighi Hospital was obtained for years 1980–2011; for the patients consecutively enrolled in our center in Bologna we used a comprehensive and strategic research plan approved by the St. Orsola-Malpighi Ethics Committee, i.e., 119/2012/U/Tess. CD diagnosis was established by positive serology and confirmatory duodenal biopsy; a genetic test (i.e., HLA-DQ2 and/or -DQ8 assessment) was performed in selected cases, i.e., when serology and histology led to contrasting results. The evaluated serological markers were: immunoglobulin A (IgA) anti-transglutaminase (TG)-2 and anti-endomysium (EmA); in cases with IgA deficiency, the immunoglobulin G (IgG) antibody anti-TG2 or against deamidated gluten peptides (DGP) were considered [9].

Biopsies have always been used as a diagnostic criterion (gold standard). From 1980 to 1990, CD diagnosis was suspected by positive anti-gliadin (AGA) antibodies and confirmed by biopsy. In the decade 1990–2000, CD was identified by AGA, EmA and anti-transglutaminase (TG) 2 IgA antibodies (the latter introduced in our center in 1997) and then again confirmed by biopsy. Genetic test was performed in selected cases, i.e., when serology and histology led to contrasting results. From 2000 to 2020, CD was detected by EmA and anti-transglutaminase (TG) IgA antibodies as well as by deamidated gliadin peptides (DGP) antibodies of IgG class followed by biopsy. Likewise to the 1990–2000 period, in the time frame 2000–2020 genetic tests were performed only in selected cases (i.e., those with incongruence between serology and histology—as above). AGA IgA testing has been dismissed since 2000 because of their well-known limits, being used only to substantiate the diagnosis of cases with suspected non-celiac gluten sensitivity.

**Figure 1:**
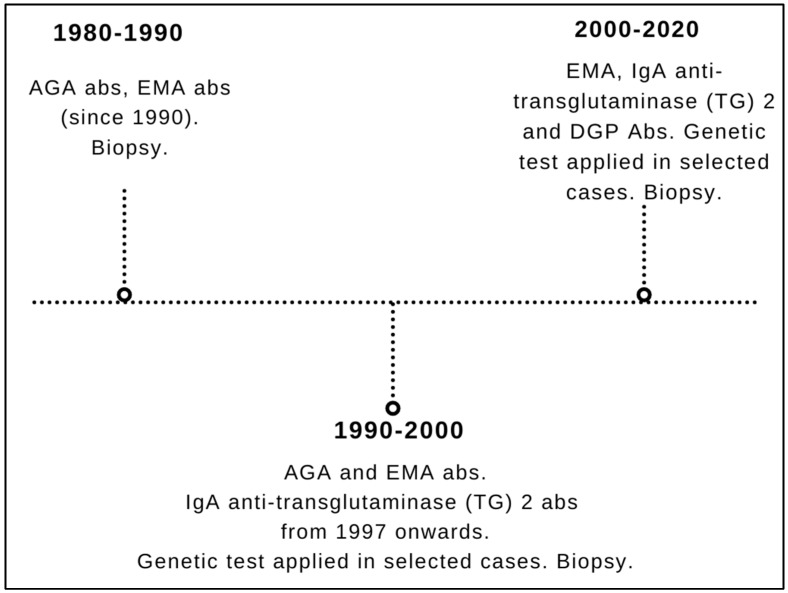
**Timeline of the different diagnostic strategies for celiac disease during the entire study timeframe (1980–2020).** AGA: anti-gliadin; EmA: anti-endomysium; DGP: deamidated gliadin peptides.

Figure 1 describes a detailed timeline of the different diagnostic procedures from 1980 to 2020.

Marsh-Oberhüber criteria [10] were applied to classify the small intestinal histopathology (n = 6 samples) taken from the bulb (n = 2) and the second duodenal portion (n = 4). The histological marker of CD was a more or less severe villous atrophy (mild 3A, partial 3B and subtotal 3C). Based on histological and serological findings, CD was classified as follows: seropositive CD (villous atrophy and positive serology), and seronegative CD (negative serology, positive HLA-DQ2/DQ8, villous atrophy recovering after 1 year of gluten-free diet—GFD). Moreover, patients with serological and genetic positivity along with normal or minimal intestinal lesions (increased number of intra-epithelial lymphocytes—IEL—and crypt hyperplasia (Marsh 0–2) were classified as potential CD.

According to the Oslo classification, the following CD phenotypes were categorized: (1) classical characterized by malabsorption syndrome (i.e., diarrhea and weight loss regardless of extra-intestinal manifestations); (2) non-classical with evidence of gastrointestinal symptoms, except from diarrhea, and extraintestinal manifestations; and (3) subclinical indicating clinically asymptomatic cases or with symptoms below the threshold of detection [2].

CD patients were investigated for gastrointestinal symptoms, dermatitis herpetiformis (DH), as well as other conditions associated with CD (e.g., osteopenia/osteoporosis, hyposplenism, microcytic [or, to a lower extent, macrocytic] anemia, infertility/recurrent miscarriages, aphthous stomatitis, hypertransaminasemia, enamel abnormalities and peripheral neuropathies) and were followed up for the occurrence of complications (i.e., type 1 and type 2 refractory celiac disease, small bowel adenocarcinoma, ulcerative jejuno-ileitis, enteropathy-associated T-cell Lymphoma, and non Hodgkin’s lymphoma) [11]. The diagnostic criteria of hyposplenism were: (1) detection of Howell-Jolly bodies in erythrocytes using classic microscopic analysis of the peripheral blood smear; (2) presence of pitted cells by phase contrast microscopy; and (3) reduced splenic dimension (<10 and <8 cm of major and minor axes, respectively) on abdominal ultrasound [12]. Extra-intestinal manifestations, complications and mortality rate were also evaluated.

### Statistical Analysis

The continuous variables were presented through mean and standard deviation (SD), while the dichotomous ones through frequency and percentage. The baseline characteristics of the enrolled patients were compared according to sex, CD type, phenotype, histotype, and presence of osteopenia, using the Student *t*-test and Chi-Squared test when appropriate.

The association of baseline characteristics with the presence of osteopenia was assessed through multivariable logistic regression analysis and expressed as odds ratio (OR) and 95% Confidence Interval (95% CI). The first analysis (Model 1) was unadjusted, while Model 2 was adjusted for the potential confounders (age and sex). A *p* value < 0.05 was considered statistically significant. Statistical analyses were performed using the R 3.5.0 software.

## 3. Results

### 3.1. Clinical Data

The distribution of CD presentation across four decades, from 1980 to 2020, is reported in Table 1. CD was diagnosed in 108 out of 1547 patients (7.0%), 203 (13.1%), 750 (48.5%) and 486 (31.4%) in the four decades, namely 1980–1989; 1990–1999; 2000–2009; and 2010–2020, respectively. Moreover, before the year 2000 the diagnoses of CD were 311 (4.5% subclinical, 45.7% classical and 49.8% non-classical) whereas afterwards they were 1236 (12.0% subclinical, 21.3% classical and 66.7% non-classical).

In particular, in the period 1980–1989 classical CD was the most commonly diagnosed phenotype (59.3%), followed by non-classical and subclinical (37.9% and 2.8%, respectively), whereas in the years 1990–1999, the most frequent clinical phenotype was the non-classical (56.2%), followed by classical (38.4%) and subclinical (5.4%). In the intervals 2000–2009 and 2010–2020 the most predominant phenotypes were non-classical phenotype (64.7% and 69.7%, respectively) followed by classical (24.4% and 16.5%) and finally subclinical (10.9% and 13.8%) (Table 1).

The main characteristics of the analyzed CD population were reported in Table 2. The median age at the diagnosis was by thirty years of age in all the decades (30 and 33 years in 1990–1999 and 2010–2020, respectively, and 34 years in the other two decades).

The majority of the patients showed seropositive (93.1%), followed by potential (5.7%) and seronegative (1.2%) CD. The analysis of phenotypes during the forty years revealed that the CD onset was symptomatic in 1384 patients (89.5% of the total, non-classical 63.3%, classical 26.2%), whereas the remaining 163 showed a subclinical phenotype (10.5%).

The most frequent histotype identified at diagnosis was Marsh 3C, found in 682 patients (44.1%), whereas the least frequent were non-atrophic lesions Marsh 0–2 (93 patients, 6%) compatible with potential CD. At diagnosis, the most frequent phenotype was non-classical (979 patients, 63.3%).

DH was found in 1.4% of the patients and IgA deficiency was detected in 59 subjects (3.81%), whereas complications were recorded in 1.8% of patients. As established by consistent literature, there is a tight correlation between CD and neurological manifestations. In our study, we found peripheral neuropathy (i.e., small fiber neuropathy, one of the most common neurological disorders in celiac patients), cerebellar ataxia, and cryptogenic epilepsy (unknown cause) in 3.2% of the investigated patients. Osteopenia was the most frequent CD-associated disorder (59.9%). In the follow-up period, equal to 11.74 (±7.85) years, 30 patients died (1.9% of the total). Although it was not possible to perform a statistical analysis of the causes of death due to missing data/incomplete data entry, the most frequent causes of mortality were ascribable to cardiovascular diseases (20 cases), while 10 patients died because of complications related to CD.

Gastrointestinal symptoms were identified in about half (n = 789; 51%) of the whole cohort. Among gastrointestinal manifestations, the most common included bloating (28%), diarrhea (24.3%), aphthous stomatitis (19.7%), alternating bowel habit (18.5%), constipation (14.1%) and gastroesophageal reflux disease (GERD, 13.5%) (Table 2).

### 3.2. Analysis by Sex

The analysis revealed that seropositive CD was predominant in both sex (91.8% and 93.5% in males and females, respectively, *p* = 0.495) (Table 3). A female preponderance was found in classical phenotype (26.9% and 23.9% of females and males, respectively; *p* < 0.001), non-classical forms (64.4% and 59.7% in females and males, respectively; *p* < 0.001), and 3C histotype (46.1% vs. 37.9%, respectively; *p* < 0.05). Among CD related manifestations, osteopenia was more frequent in females (64.3% vs. 46.2%; *p* < 0.001). Also, long-term follow-up revealed statistically significant differences between females and males (11.97 ± 7.95 years in females vs. 11.01 ± 7.48 years in males, respectively; *p* < 0.05). Markedly, sex did not influence the age at the time of diagnosis. Furthermore, there were no statistically significant differences between sex regarding the IgA deficiency, and death.

### 3.3. Analysis by CD Type

The analysis of CD type (Table 4) reveals that seronegative CD patients show a higher mean age at diagnosis than those with seropositive and potential forms (45.74 ± 12.52 vs. 33.04 ± 12.40 vs. 32.56 ± 14.41 years, respectively; *p* < 0.001). Moreover, in seronegative patients, the classical phenotype and 3C histotype occurred more frequently than the seropositive and potential groups (phenotype: 73.7% vs. 26.8% vs. 4.5%, respectively; *p* < 0.001—histotype: 84.2% vs. 46.3% vs. 0%, respectively; *p* < 0.001). Notably, the most common phenotype in patients with seropositive and potential forms was non-classical one (both 63.7%). The 94.7% of patients with seronegative CD had osteopenia as associated disorder (*p* < 0.001) as well as a higher frequency of hyposplenism (15.8%; *p* < 0.001) and neurological disorders associated with CD (neuroCD) (17.6%; *p* = 0.002) than the other two groups.

Finally, patients with seronegative CD reported a higher risk of complications (21.1%; *p* < 0.001) and death (10.5%; *p* = 0.021), whereas the seropositive type was associated with a longer follow-up compared with the seronegative and potential groups (12.04 ± 7.87 vs. 7.95 ± 7.85 and 7.61 ± 5.95 years, respectively; *p* < 0.001).

### 3.4. Analysis by Phenotype

Data showed a statistically significant correlation between age and type of disease onset, in particular the classical form reported the highest mean age between the three phenotypes (classical = 36.12 ± 13.74 vs. non-classical = 32.36 ± 11.92, vs. subclinical = 30.16 ± 12.33 years; *p* < 0.001) (Table 5).

In women the classical form prevails, followed by non-classical and subclinical (classical 77.8% vs. non-classical 77.0% vs. subclinical 62.0%; *p* < 0.001).

Regarding the CD type, the seropositive was the most represented in all the phenotypes (82.8% in subclinical, vs. 95.6% in classical, vs. 93.8% in non-classical; *p* < 0.001). The histotype 3C was the most common in both classical and non-classical forms (80.7% vs. 34.7%, respectively; *p* < 0.001), while the 3A histotype was most common in subclinical CD (54.6%). The analysis of CD associated disorders showed that osteopenia and hyposplenism were more frequently associated with classical phenotype (classical 87.7% vs. non-classical 55.0% vs. subclinical 20.2%; *p* < 0.001—classical 5.9% vs. non-classical 0.6% vs. subclinical 0.0%, respectively; *p* < 0.001). Also, the majority of patients who died at follow-up exhibited the classical form (classical 5.2% vs. non-classical 0.6% vs. subclinical 1.8%; *p* < 0.001).

### 3.5. Serological Tests

IgA anti-TG2 positivity was found in 1469 out of 1547 (95%), of whom 93% were positive for both IgA EmA and anti-TG2. Seventy-eight patients were negative for IgA anti-TG2 and EmA; of this subset, 59 patients showing a selective IgA deficiency, tested positive for IgG anti-TG2 or DGP. Seronegative subjects were 1.2% of the total (19 out of 1547, 13 females) and showed a marked prevalence of classical phenotype (14; *p* < 0.001). Seronegative CD patients were diagnosed significantly later than seropositive ones (46 vs. 33 years; *p* < 0.005). Seven seronegative subjects were positive for IgG anti-gliadin antibodies (AGA).

### 3.6. Extraintestinal Manifestations

**Figure 2:**
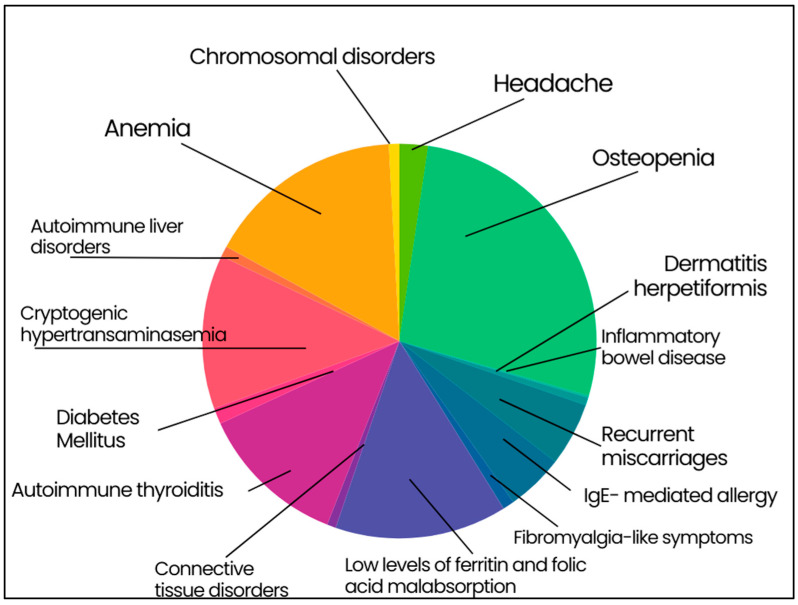
**Extraintestinal manifestation in enrolled celiac patients**. Osteopenia 59.9%; anemia 35.8%; cryptogenic hypertransaminasemia 27.9%; recurrent miscarriages 11.5%; headache 5.2%; fibromyalgia-like symptoms 2.1%; IgE-mediated allergy 10.4%; low levels of ferritin and folic acid malabsorption 31.2%; autoimmune thyroiditis 26.9%; type 1 diabetes mellitus 2.9%; dermatitis herpetiformis 1.4%; autoimmune liver disorders 1.9%; connective tissue disorders 1.6%; chromosomal disorders 1.9%; inflammatory bowel disease 0.4%.

The most frequent extra-intestinal manifestation was osteopenia (926 out of 1547 patients, 59.9%) associated with 25-OH Vitamin D3 low levels in patients with the classical CD (*p* < 0.001). Other extra-intestinal manifestations included anemia (35.8%), cryptogenic hypertransaminasemia (27.9%), recurrent miscarriages (11.5%), headache (5.2%), and fibromyalgia-like symptoms (2.1%). IgE mediated allergy was present in the 10.4% of patients, often associated to positivity for specific IgE to *Graminaceae* and mites. With regard to the 87% of patients with anemia, they showed low levels of ferritin and folic acid malabsorption (482 subjects, 31.2% of the total population). A subset of CD patients (26.9%) had autoimmune thyroiditis (hypothyroidism was present in 13.1% of cases). DM1 was found in 2.9% and DH was detectable in 1.4% of patients. Autoimmune liver disorders (e.g., primary biliary cholangitis and autoimmune hepatitis) affected 1.9%, whereas connective tissue disorders (i.e., Sjögren syndrome and systemic sclerosis) were diagnosed in 1.6% of patients. Finally, chromosomal disorders affected 1.9% of patients (i.e., 24 Down and 5 Turner syndromes) and inflammatory bowel diseases were found in 6 patients (0.4%; 2 Crohn’s disease and 4 ulcerative colitis) (Figure 2).

### 3.7. Gastrointestinal Symptoms in Patients on GFD

**Figure 3:**
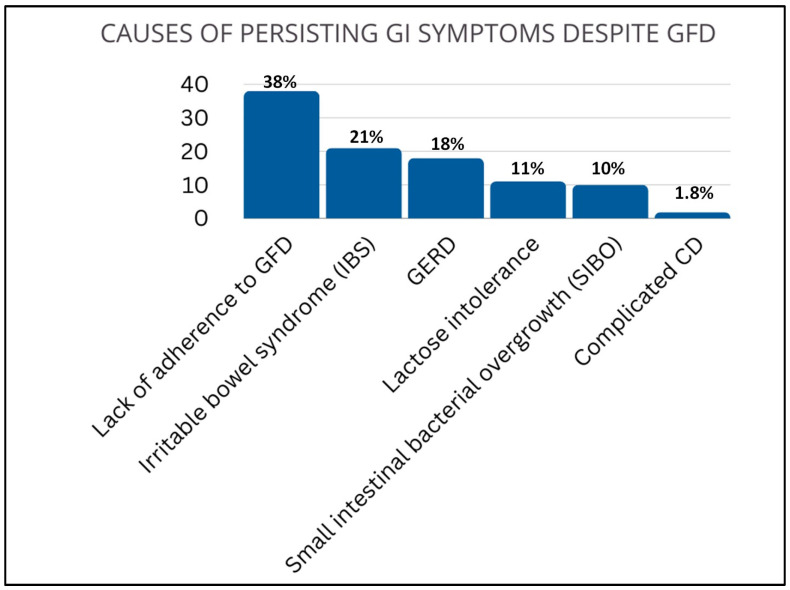
**Causes of persisting GI symptoms despite GFD.** Lack of adherence to GFD (38%), irritable bowel syndrome (IBS) (21%), gastroesophageal reflux disease GERD (18%), lactose intolerance (11%), small intestinal bacterial overgrowth (SIBO) (10%), and complicated CD (1.8%).

The number of patients who regularly attended clinical and biochemical follow-up was 1266 (82%). The follow-up lasted from 18 months to 19.6 years (11.74 ± 7.85 years). GFD is the cornerstone of celiac disease treatment, however its role and actual need in patients with potential CD is debated. Data from our group and shared by the scientific community, suggested to recommend GFD only in adult cases of symptomatic potential celiac disease [13].The majority of patients had a good response to GFD (n = 1025; 81%); the remaining 241 subjects presented GI symptoms regardless gluten withdrawal. The causes leading to persisting gastrointestinal symptoms were many and included lack of adherence to GFD (38%), irritable bowel syndrome (IBS) (21%), GERD (18%), lactose intolerance (11%), small intestinal bacterial overgrowth (SIBO) (10%), and complicated CD (1.8%) (Figure 3).

### 3.8. Complications

The number of patients presenting complications was low (28 out of 1547; 1.8%). Due to the small number of the first complicated cases, a formal comparison (i.e., complicated vs. uncomplicated patients) was not statistically appropriate. Thus, a descriptive analysis of the complicated patients subgroup and mortality rate was performed. Table 6 summarizes the complications affecting CD patients: type 1 refractory CD (RCD1) was most common being detectable in 12 patients (0.8%), followed by small bowel adenocarcinoma (SBA) (0.5%), ulcerative ileitis and enteropathy-associated T-cell Lymphoma (EATL), both accounting for 0.3%. Finally, type 2 RCD (RCD2) and non Hodgkin’s lymphoma (NHL) were diagnosed in 2 patients (0.1% of the total).

**Figure 4:**
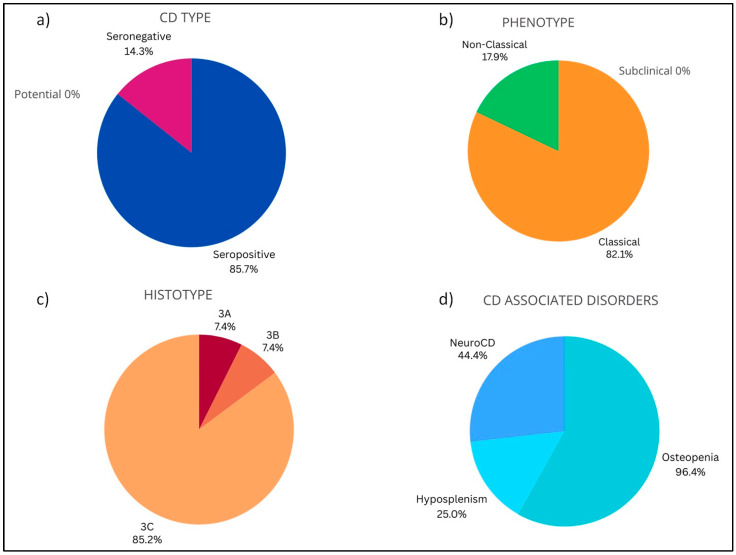
**Baseline characteristics in enrolled celiac patients with complications**. (**a**) CD type: Seropositive (85.7%), Potential (0.0%), Seronegative (14.3%). (**b**) Phenotype: Subclinical (0.0%), Classical (82.1%), Non-Classical (17.9%). (**c**) Histotype: Marsh 0–2 (0.0%), 3A (7.4%), 3B (7.4%), 3C (85.2%). (**d**) CD associated disorders: Osteopenia (96.4%), Hyposplenism (25.0%), NeuroCD (44.4%).

Complications occurred at a median age of 55 years and mainly involved females (n = 22; 78.6%). Seropositivity (85.7%), classical phenotype (82.1%) and histotype 3C (85.2%) were the most common features in complicated CD. Complications never occurred in subclinical phenotype. Moreover, 96.4% (n = 27) of the patients with complications presented osteopenia, 25% hyposplenism (n = 7), and 44.4% neuroCD (n = 8) (Figure 4). Patients with complications reported neither DH nor IgA deficiency. Ten out 28 patients died (35.7%) for complicated CD.

### 3.9. Mortality

Mortality in patients without complications was low (0.013; namely, 1.3 patients died every 100 patients without complications), and occurred at a median age of 58.73 ± 13.62 years (Table 7). The majority of patients undergone a fatal outcome were female (21 subjects; 70%), with seropositivity (90%), classical CD phenotype (70%), 3C histotype (70%) and showed osteopenia as most present CD associated disorder (96.7%) and hyposplenism in the 30% of cases (n = 9). On third of dead patients presented complication (n = 10; 33%). Deceased patients reported neither DH nor IgA deficiency.

## 4. Discussion

Celiac disease is a complex autoimmune disorder involving both adaptive and innate mechanisms, triggered by gluten ingestion in genetically predisposed subjects [14]. CD patients are at risk of complications (e.g., osteoporosis and intestinal lymphoma). However, despite improvements in the diagnosis of CD, most patients still experience misdiagnosis or a substantial delay in diagnosis. Thus, awareness of the spectrum of manifestations of CD can favor early diagnosis and improve therapy [15]. This study analyzed the clinical, serological, and histopathological features of a large cohort of CD patients consecutively diagnosed in a single Italian referral center in 40 years of clinical activity. Our analysis did not consider first-degree relatives of celiac patients. Indeed, it has been already established by published data that the risk percentage for first-degree relatives ranges from 1.6 to 38% [16]. Our analysis revealed that the rate of diagnoses increased over time. In the last 20 years (2000–2020), the number of CD diagnoses quadruplicated going from 311 (in the previous two decades—1980–2000) to 1236, a finding in line with Catassi et al. who reported a fivefold incidence of CD in the last 25 years [17]. However, compared to 2000–2010 decade, the number of new CD diagnoses in the last decade decreased in our referral center. A possible explanation is the reduction in case finding for CD in the 2010–2020 period after an intensive search in at-risk groups carried out in the previous decade. Furthermore, COVID-19 pandemia, involving Italy since early 2020, may have hindered patients’ referrals to various centers including ours.

In agreement with previously published data by our group [7] and others [18], CD onset changed over the years showing on the one hand a considerable reduction of the classical phenotype (from 59.3% in the first 10 years to 16.5% in the last 10 years) and on the other hand an increase of non-classical and subclinical phenotypes detected in 83.5% of cases in the last ten years. CD was diagnosed mainly in females, with a female/male ratio of 3.5:1, according to our previous study and other well-established data [19]. The median age at diagnosis was the early thirties, however, in a small subset (about 3%) the disease appeared in elderly (>65 years) patients indicating that CD can manifest at any age of life, as previously reported [20]. Likely, the modern serological screening—i.e., anti-TG2, EMA, and DGP—significantly contributed to identify CD in its preclinical stage or even in apparently asymptomatic cases [21,22]. The majority of patients (63.3%) showed the non-classical CD, with atypical gastrointestinal and extraintestinal manifestations, while the 10% had the subclinical form characterized by villous atrophy in the absence of typical symptoms. Notably, an interesting finding emerged by this analysis was that subclinical CD were predominantly male with a relative percentage almost doubling that of females.

The classical form was present in more than a quarter of patients (26%) who complained of the typical gastrointestinal manifestations (i.e., diarrhea and malabsorption). Diarrhea should be no longer considered one of the primary symptoms of CD [7,22]. According to data by Lebwohl and Rubio-Tapia, the percentage of CD patients showing diarrhea markedly decreased from 73% before 1993, when serologic tests were introduced in the clinical practice, to 43% afterwards. The reason for this drop in diarrhea frequency can be explained with the greater attention paid by physicians to other symptoms/signs commonly thought not to be related to CD, such as IBS-like manifestations (e.g., bloating/irregular bowel habit), osteoporosis, anemia, infertility, migraines, neuropsychiatric disorders, and liver enzyme abnormalities [7,22,23,24,25,26,27]. Thus, the diagnosis of CD based on extraintestinal manifestations collectively outnumber the diagnoses driven by the occurrence of diarrhea, thus making the presentation with diarrhea no longer definable as “typical” CD.

More than 90% of patients were labeled as seropositive CD exhibiting both autoantibodies and villous atrophy. In contrast, about 6% of patients showed potential CD, identified by serological screening with positivity for HLADQ2/DQ8 and normal intestinal mucosa or minimal lesions characterized by an increased number of IELs with or without crypt hyperplasia. Finally, a small subgroup of patients (1.2%) showed seronegative CD. The negative serological profile correlated to specific aspects. First, almost three-quarters (73.7%) of patients with seronegative CD showed a classical phenotype and the vast majority (84.2%) had severe (3C) atrophy. This trend indicates that small bowel biopsy should always be performed in patients with malabsorption to rule out CD diagnosis, despite a negative serology. Secondly, seronegative CD patients were mainly females (68.4%), with a higher median age at the diagnosis (45 years) compared to seropositive CD (33 years), a finding which confirmed and expanded our previous study [7]. Finally, seronegative CD was found to be closely associated with osteopenia (94.7% of the total) and, to a minor, though statistically significant, extent with hyposplenism (15.8% seronegative, vs. 1.9% and 0% of seropositive and potential, respectively). Also, more than one-fifth (21.1%) of seronegative patients showed complications that were absent in potential CD patients and very low in patients with seropositive CD (1.7%).

The evaluation of histotype revealed severe intestinal damage (type 3B, 3C) only in 71% of cases, a result in contrast with previous studies [28], but in line with our previous data [7]. Type 3A, indicating partial atrophy, was found in 23.1% of the CD population, in line with published data [7,28,29].

The analysis by clinical phenotype showed that the classical form occurred at a median age of 36 years (later than the subclinical and the non-classical CD which were diagnosed at a median age of 30 and 32 years, respectively), had a seropositive profile (96% of cases) and highly correlated to severe mucosa damages (3C; 80.7%) and to a higher risk of death than the other two phenotypes (5.2% vs. 1.8 of subclinical and 0.6 of non-classical). Both classical and non-classical CD showed osteopenia (87.7% and 55%, respectively; *p* < 0.001).

Among CD-associated disorders, osteopenia was highly frequent in our cohort (almost 60% of cases). As expected, analysis by sex confirmed that females were more prone to osteopenia than males (64.3 vs. 46.2%, respectively; *p* < 0.001). These data expanded our previous findings showing that 52% of the two-thirds of the 770 analyzed CD patients had osteopenia/osteoporosis [7]. This condition, which results from malabsorption of calcium and 25-OH Vitamin D3, is a typical feature of patients with CD. The prevalence of osteoporosis in the world is 23.1% (95% CI 19.8–26.9) in women and 11.7% (95% CI 9.6–14.1) in men [30]. Our recent review reported that osteoporosis is estimated to affect 18–35% of CD population [31,32,33,34]. Two studies independently found a more severe form of low mineral bone density in men than pre-and post-menopausal women, suggesting a protective role of estrogens in women [31,35]. However, in CD female sex is considered at higher risk of osteoporosis for indirect (early menopause, amenorrhea) and direct effects (malabsorption).

Besides osteopenia, extra-intestinal manifestations included a vast range of conditions including anemia, cryptogenic hypertransaminasaemia, headache, fibromyalgia-like symptoms, IgE-mediated allergy. Data confirmed that CD is frequently associated with low levels of ferritin and folic acid resulting from intestinal malabsorption with the latter contributing to an increased risk of miscarriages. A correlation between CD and autoimmune diseases was also established, as previously reported [7]. This relationship can be due to the ubiquitary distribution of TG2 in organs other than the small bowel. We confirmed the presence of autoimmune thyroiditis (Hashimoto Thyroiditis) in about one-fourth of CD patients, half of whom subsequently developed clinical hypothyroidism. The percentage of participants showing DH was 1.4% and the presence of type 1 DM1 in our series reflected the rates previously reported in literature (2.9%) [36]. The low percentage of type 1 Diabetes Mellitus (DM) in CD patients on GFD further proved that gluten avoidance could exert a protective role of type 1 DM onset [36]. Brain and liver diseases are commonly associated with CD [37,38]. Connective tissue disorders, such as Sjögren syndrome and systemic sclerosis were found associated with CD [39,40]. Among chromosomal disorders in our series, Down and Turner syndrome confirmed to be the most represent chromosomal abnormality in patients with CD.

In this analysis we also evaluated the effect of GFD on 1226 CD patients, followed up for a period ranging from 18 months to 19.6 years. We found that about 20% of patients who declared to follow a GFD reported GI symptoms despite gluten avoidance. This is in line with our previous results [7], and the reasons underlying this phenomenon should be ascribed mainly to the lack of compliance to GFD (mainly due to inadvertent food contamination) and functional gastrointestinal disorders (e.g., irritable bowel syndrome, GERD, and lactose intolerance). In our case, we exclude that persistent GI symptoms depend on CD misdiagnosis, as our population was diagnosed according to well-established criteria. The persistency of GI symptoms despite GFD has also been described by a Swedish study investigating 524 diagnosed celiac subjects, of which about 40% of participants reported GI symptoms persistency despite gluten avoidance [41].

In our population, complications occur in 1.8% of cases, including type 1 refractory CD, small bowel adenocarcinoma, ulcerative jejuno-ileitis, enteropathy-associated T-cell lymphoma, type 2 refractory CD and non-Hodgkin’s lymphoma. These data nearly double previous studies [11,41] and our former analysis that registered 0.9% of complicated patients [7]. Complications often occurred in CD patients with diarrhea and malabsorption, HLA-DQ2 homozygosis, and delayed diagnosis [42,43,44].

However, these data confirm the most recent estimate of complication rate [11,45], which attests the percentage of about 1% at variance with early projections, which predicted a complication rate of 5–10%, overestimating the actual trend.

Data on this large sample size confirmed our previous results [7]: (i) the better sensitivity of IgA anti-TG2 compared to IgA EmA for the diagnosis of CD; (ii) IgA EmA positivity was always found in patients with IgA anti-TG2; (iii) about two-thirds of subjects who tested negative for IgA TG2/EmA also had IgA deficiency (while being positive for IgG anti-TG2/DGP); and (iv) only a small subgroup of patients had seronegative CD (1.2%).

## 5. Conclusions

Despite possible limitations of this study, i.e., its retrospective nature, which could have biased the data analysis and the lack of information (for example on the pediatric CD population which has been not investigated), our analysis contained important data. Specifically, a large cohort of CD patients was thoroughly investigated and clinical presentation, serology, histology, associated disorders, complications, and mortality rate were assessed over a long (40-year) follow-up period. The results of this study confirmed and broadened previous knowledge on CD. In conclusion, we showed that: (i) according to the recently established criteria, CD clinical profile changed with a prevalence of non-classical and subclinical phenotypes vs. classical forms; (ii) the serological screening allowed an ‘early diagnosis’ of CD cases by identifying the disease at subclinical stage, thus contributing to explain the increase in the number of diagnosis; (iii) some CD-related disorders correlated with sex, e.g., osteopenia being more prevalent in women, a finding prompting faster and effective management.

## Figures and Tables

**Table 1:** Distribution of CD presentation across the four decades analyzed (1980–2020).

Period	Classical	Non-Classical	Subclinical	Total
1980–1989	64 (59.3%)	41 (37.9%)	3 (2.8%)	108 (7.0%)
1990–1999	78 (38.4%)	114 (56.2%)	11 (5.4%)	203 (13.1%)
2000–2009	183 (24.4%)	485 (64.7%)	82 (10.9%)	750 (48.5%)
2010–2020	80 (16.5%)	339 (69.7%)	67(13.8%)	486 (31.4%)

**Table 2:** Baseline characteristics in enrolled celiac patients.

		Overall Sample N = 1547
**Age (years), mean (SD)**		33.11 (24.4)
**Female sex, n (%)**		1170 (75.6)
**Male sex, n (%)**		337 (24.4)
**CD type, n (%)**		
*Seropositive*		1440 (93.1)
*Potential*		88 (5.7)
*Seronegative*		19 (1.2)
**Phenotype, n (%)**		
*Subclinical*		163 (10.5)
*Classical*		405 (26.2)
*Non-Classical*		979 (63.3)
**Herpetiformis dermatitis, n (%)**		21 (1.4)
**IgA deficiency, n (%)**		59 (3.81)
**Histotype, n (%)**		
	*Marsh 0–2*	93 (6.0)
	*3A*	357 (23.1)
	*3B*	414 (26.8)
	*3C*	682 (44.1)
**CD associated disorders**		
*Osteopenia*, *n (%)*		926 (59.9)
*Hyposplenism*, *n (%)*		30 (1.9)
*NeuroCD*, *n (%)*		49 (3.2)
**Gastrointestinal manifestations, n (%)**		
*Diarrhea*, *n (%)*		376 (24.3%)
*Bloating*, *n (%)*		434 (28%)
*Aphthous stomatitis*, *n (%)*		305 (19.7%)
*Alternating bowel habit*, *n (%)*		286 (18.5%)
*Constipation*, *n (%)*		218 (14.1%)
*Gastroesophageal reflux disease (GERD)*, *n (%)*		209 (13.5%)
**Complications, n (%)**		28 (1.8)
**Number of complications, mean (SD)**		0.02 (0.16)
**Follow-up (years), mean (SD)**		11.74 (7.85)
**Death, n (%)**		30 (1.9), of whom only 10 (0.65) related to CD complications

**Table 3:** Baseline characteristics in enrolled celiac patients according to sex.

		Sex		*p*-Value
		Male (n = 377)	Female (n = 1170)	
**Age (years), mean (SD)**		**33.63 (13.72)**	32.94 (12.22)	0.357
**CD type, n (%)**				0.495
	*Seropositive*	346 (91.8)	1094 (93.5)	
	*Potential*	25 (6.6)	63 (5.4)	
	*Seronegative*	6 (1.6)	13 (1.1)	
**Phenotype, n (%)**				**<0.001**
	*Subclinical*	62 (16.4)	101 (8.6)	
	*Classical*	90 (23.9)	315 (26.9)	
	*Non-Classical*	225 (59.7)	754 (64.4)	
**Herpetiformis dermatitis, n (%)**		8 (2.1)	13 (1.1)	0.223
**IgA deficiency, n (%)**		12 (3.18)	47 (4.0)	1.000
**Histotype, n (%)**				**0.029**
*Marsh 0–2*		27 (7.2)	66 (5.6)	
*3A*		102 (27.1)	255 (21.8)	
*3B*		105 (27.9)	309 (26.4)	
*3C*		143 (37.9)	539 (46.1)	
**CD associated disorders**				
*Osteopenia*, *n (%)*		174 (46.2)	752 (64.3)	**<0.001**
*Hyposplenism*, *n (%)*		5 (1.3)	25 (2.1)	0.437
*NeuroCD*, *n (%)*		11 (2.9)	38 (3.3)	0.867
**Complications, n (%)**		6 (1.6)	22 (1.9)	0.078
**Follow-up (years), mean (SD)**		11.01 (7.48)	11.97 (7.95)	**0.038**
**Death, n (%)**		9 (2.4)	21 (1.8)	0.610

**Table 4:** Baseline characteristics in enrolled celiac patients according to CD type.

		CD Type			*p*-Value
		Seropositive (n = 1440)	Potential (n = 88)	Seronegative (n = 19)	
**Age (years), mean (SD)**		**33.04 (12.40)**	32.56 (14.41)	45.74 (12.52)	**<0.001**
**Female sex, n (%)**		1094 (76.0)	63 (71.6)	13 (68.4)	0.495
**Phenotype, n (%)**					**<0.001**
	*Subclinical*	135 (9.4)	28 (31.8)	0 (0.0)	
	*Classical*	387 (26.8)	4 (4.5)	14 (73.7)	
	*Non-Classical*	918 (63.7)	56 (63.6)	5 (26.3)	
**Herpetiformis dermatitis, n (%)**		18 (1.2)	3 (3.4)	0 (0.0)	0.207
**IgA deficiency, n (%)**		59 (4.1)	0 (0.0)	-	0.830
**Histotype, n (%)**					**<0.001**
	*Marsh 0–2*	5 (0.3)	88 (100.0)	0 (0.0)	
	*3A*	355 (24.7)	0 (0.0)	2 (10.5)	
	*3B*	413 (28.7)	0 (0.0)	1 (5.3)	
	*3C*	666 (46.3)	0 (0.0)	16 (84.2)	
**CD associated disorders**					
	*Osteopenia*, *n (%)*	890 (61.8)	18 (20.5)	18 (94.7)	**<0.001**
	*Hyposplenism*, *n (%)*	27 (1.9)	0 (0.0)	3 (15.8)	**<0.001**
	*NeuroCD*, *n (%)*	45 (3.1)	1 (1.1)	3 (17.6)	**0.002**
**Complications, n (%)**		24 (1.7)	0 (0.0)	4 (21.1)	**<0.001**
**Follow-up (years), mean (SD)**		12.04 (7.87)	7.61 (5.95)	7.95 (7.85)	**<0.001**
**Death, n (%)**		27 (1.9)	1 (1.1)	2 (10.5)	**0.021**

**Table 5:** Baseline characteristics in enrolled celiac patients according to phenotype.

	Phenotype			*p*-Value
	Subclinical (n = 163)	Classical (n = 405)	Non-Classical (n = 979)	
**Age (years), mean (SD)**	30.16 (12.33)	36.12 (13.74)	32.36 (11.92)	**<0.001**
**Female sex, n (%)**	101 (62.0)	315 (77.8)	754 (77.0)	**<0.001**
**CD type, n (%)**				**<0.001**
*Seropositive*	135 (82.8)	387 (95.6)	918 (93.8)	
*Potential*	28 (17.2)	4 (1.0)	56 (5.7)	
*Seronegative*	0 (0.0)	14 (3.5)	5 (0.5)	
**Herpetiformis dermatitis, n (%)**	1 (0.6)	1 (0.2)	19 (1.9)	0.032
**IgA deficiency, n (%)**	0 (0.0)	24 (5.6)	35 (3.6)	0.637
**Histotype, n (%)**				**<0.001**
*Marsh 0–2*	28 (17.2)	4 (1.0)	61 (6.2)	
*3A*	89 (54.6)	10 (2.5)	258 (26.4)	
*3B*	30 (18.4)	64 (15.8)	320 (32.7)	
*3C*	16 (9.8)	327 (80.7)	339 (34.7)	
**CD associated disorders**				
*Osteopenia*, *n (%)*	33 (20.2)	355 (87.7)	538 (55.0)	**<0.001**
*Hyposplenism*, *n (%)*	0 (0.0)	24 (5.9)	6 (0.6)	**<0.001**
*NeuroCD*, *n (%)*	1 (0.6)	13 (3.3)	35 (3.6)	0.137
**Complications, n (%)**	0 (0.00)	23 (5.7)	5 (0.5)	**<0.001**
**Follow-up (years), mean (SD)**	9.10 (6.07)	14.84 (9.02)	10.89 (7.20)	**<0.001**
**Death, n (%)**	3 (1.8)	21 (5.2)	6 (0.6)	**<0.001**

**Table 6:** Complications in enrolled celiac patients.

	Patients with Complications (n = 28)
**RCD type 1, n (%)**	12 (42.9) [0.8] *
**RCD type 2, n (%)**	2 (7.14) [0.1] *
**EATL, n (%)**	4 (14.3) [0.3] *
**NHL, n (%)**	2 (7.14) [0.1] *
**SBA, n (%)**	8 (28.57) [0.5] *
**Ulcerative ileitis, n (%)**	4 (14.29) [0.3] *

Legend: RCD = Refractory Celiac Disease, EATL = Enteropathy-associated T-cell Lymphoma, NHL = Non-Hodgkin’s Lymphoma, SBA = Small Bowel Adenocarcinoma. * [ ] = % on the overall population.

**Table 7:** Baseline characteristics in enrolled celiac died patients.

		Deaths (n = 30)
**Age (years), mean (SD)**		58.73 (13.62)
Female sex, n (%)		21 (70.0)
CD type, n (%)		
	*Seropositive*	27 (90.0)
	*Potential*	1 (3.3)
	*Seronegative*	2 (6.7)
Phenotype, n (%)		
	*Subclinical*	3 (10.0)
	*Classical*	21 (70.0)
	*Non-Classical*	6 (20.0)
Histotype, n (%)		
	*Marsh 0–2*	1 (3.4)
	*3A*	5 (16.6)
	*3B*	3 (10)
	*3C*	21 (70)
CD associated disorders		
*Osteopenia*, *n (%)*		29 (96.7)
*Hyposplenism*, *n (%)*		9 (30.0)
*NeuroCD*, *n (%)*		5 (18.5)
Complications, n (%)		10 (33.3)
Follow-up (years), mean (SD)		12.20 (11.77)

## Data Availability

The datasets analyzed during the current study are not publicly available due to privacy reason but are available from the corresponding author on reasonable request.

## Abbreviations

CD	Celiac Disease
HLA	Human leukocyte antigen
DGP	Deamidated gliadin peptide
IgA	Immunoglobulin A
TG	Transglutaminase
EmA	Anti-endomysium;
GFD	Gluten-free diet
DH	Dermatitis herpetiformis
OR	Odds ratio
CI	Confidence Interval
GERD	Gastroesophageal reflux disease
neuroCD	Neurological disorders associated with CD
AGA	Anti-gliadin antibodies.
